# Dithiosalicylate of Soda

**Published:** 1891-03-28

**Authors:** 


### THERAPEUTICS.

### DITHIOSALICYLATE OF SODA.

An extensive trial of this substance in various cases, chiefly of a rheumatic nature, has led us to regard it as a valuable addition to our therapeutic resources. Two isomeric substances, named Dithiosalicylate I. and II., have been prepared by Baum, having the following chemical constitution:—

$$\begin{array}{l} S{-}C_6\,H_3\begin{array}{l}-OH\\-COONa\end{array}\\ |\\ S{-}C_6\,H_3\begin{array}{l}-COONa\\-OH\end{array}\end{array}$$

It is therefore two atoms of sulphur bound to two molecules of salicylate of soda. On the addition of an acid, yellow viscid drops of dithiosalicylic acid are precipitated by the combination of the sodium atoms with the added acid. It is a very hygroscopic greyish-white powder, rapidly liquefying if exposed to the atmosphere, and is entirely dissolved by a small proportion of water.

It has been clinically investigated by Lindenborn and Hueppe, and has been introduced to this country by Messrs. Burroughs and Wellcome. Lindenborn failed to discover any trace of salicylic acid or dithiosalicylic acid in the urine of a patient to whom he had administered the drug for eight days.

As regards its germicidal powers, Hueppe's researches showed that it was able to destroy anthrax spores in about forty-five minutes, the salicylate of soda not possessing any such action. Similar results were obtained with the bacteria of cholera and enteric fever, staphylococcus aureus, and the pyocyaneus, or blue pus microbe.

These observers seem to have had most pleasing results with the dithiosalicylate in acute rheumatism with high temperature, and in gonorrheal rheumatism. Lindenborn states that he finds that it has the following advantages over the salicylate: (1) It has a powerful action and need not be administered in such large doses as the salicylate. (2) It does not give rise to gastric disturbance, nor does it have any deleterious action on the heart. (3) It does not produce collapse. (4) Tinnitus aurium was not observed in any of his cases.

In cases of acute rheumatism, with effusion in several joints and high temperature, we have found it decidedly less efficacious than the salicylate. Hueppe, on the other hand, observed a reduction of two or three degrees in the evening temperature followed the administration of two to four doses of three grains each. In a case of acute rheumatism under our care, with temperature of 100·5, pulse 120, pain and swelling in both knees, and ankles and right hip joint, the patient had been confined to bed for three days, was, when seen, was getting worse. The joints were wrapped in cotton wool, and a three-grain pill of the dithiosalicylate given every six hours for two days without the slightest relief. He was then put on twenty grains of the salicylate every six hours. Marked relief was obtained after two doses, and he continued to improve rapidly, being free from all acute symptoms in two days. This case fairly represents our experience in many others of a similar character. On the other hand, we have found that the dithiosalicylate, when given in doses of three grains thrice daily, relieves the pain and the symptoms generally of the more chronic cases far more effectually than the salicylate, and there appears to be a less tendency to relapse after its use.

We can also confirm Hueppe's experience in gonorrheal rheumatism, the pains, especially in some of our chronic cases, being relieved when every other remedy had failed. The best form of administration is in pills. These must be varnished, or at any rate coated with some material impervious to moisture, otherwise the pills rapidly liquefy.

